# Risk factors for postpartum depression in women undergoing elective cesarean section: A prospective cohort study

**DOI:** 10.3389/fmed.2022.1001855

**Published:** 2022-09-28

**Authors:** Rong Lin, Yan Lu, Wei Luo, Bing Zhang, Zhiqiang Liu, Zhendong Xu

**Affiliations:** ^1^Department of Anesthesiology, Shanghai First Maternity and Infant Hospital, School of Medicine, Tongji University, Shanghai, China; ^2^Clinical and Translational Research Center, Shanghai First Maternity and Infant Hospital, School of Medicine, Tongji University, Shanghai, China

**Keywords:** cesarean section, postpartum depression, prenatal anxiety, prospective study, Edinburgh Postnatal Depression Scale

## Abstract

**Background:**

Postpartum depression (PPD) has adverse effects on maternal and child health. Cesarean section (CS) is suggested to be associated with PPD, but no study has examined the risk factors for PPD in women who underwent CS. Therefore, this study aimed to investigate this association.

**Methods:**

A prospective observational study was conducted between December 2020 and September 2021. In total, 590 women who underwent elective CS participated in this study. Data were collected using a questionnaire through a face-to-face interview at three time points: 32nd week of gestation, 2 days postpartum, and 6 weeks postpartum. PPD was defined by an Edinburgh Postnatal Depression Scale (EPDS) score of ≥ 11 at 6 weeks postpartum. Multivariate logistic regression analysis was performed to identify the risk factors for PPD.

**Results:**

Among the 590 women, 25.4% had PPD (142/590). After adjustment for the confounding factors, high antenatal self-rating anxiety scale score (OR = 1.10, 95% CI = 1.04–1.16), PPD symptoms (EPDS ≥ 11) at 2 days postpartum (OR = 6.17, 95% CI = 1.35–28.31), and pain at 6 weeks postpartum (OR = 2.14, 95% CI = 1.24–3.69) were independently associated with PPD.

**Conclusion:**

Prenatal anxiety, PPD symptoms occurring at an early postoperative stage, and pain at 6 weeks postpartum may be associated with an increased risk of PPD among women who undergo CS.

## Introduction

Postpartum depression (PPD) is a serious public health issue worldwide. PPD generally occurs 4–6 weeks after childbirth, with symptoms including depressed mood, restlessness, irritability, or anxiety, loss of interest or pleasure, sleep disturbance, and expression of little interest in baby (1). The prevalence of PPD varies from 7.6 to 39% in different countries studied using self-report questionnaires (2). In China, more than 16% of women develop depression during pregnancy and after childbirth (3). PPD has adverse effects on both mothers and children, increase the risk of self-harm or even suicide, and impair maternal-infant bonding, leading to emotional and cognitive disorders in children (1, 4). Therefore, knowledge of the risk factors for PPD has great implications and may help identify mothers at high risk who may require intervention.

Previous studies have identified several potential risk factors including psychosocial and medical factors, such as anxiety, prenatal depression or history of depression, and maternal and infant complications (5, 6). Obstetric risk factors, such as induced labor, placenta previa, and mode of delivery, have also been reported (7). As cesarean section (CS) rates continue to increase globally, there is a growing concern regarding the association between CS and PPD (8, 9).

CS is one of the most common surgeries performed worldwide. The global average rate of CS has been reported to be 21.1%, and the rate of elective CS is also increasing (10). According to a report by the World Health Organization, the CS rate in China reached 46% during the period from 2007 to 2008, ranking first in the world (11). CS may increase the risk of maternal and neonatal morbidity and lead to waste of human and financial resources (12). Currently, it is believed that CS and emergency CS are associated with the increased risk of PPD (8, 13, 14).

Many studies have evaluated the relationship between CS and PPD among cesarean and vaginally delivered women (8, 15). However, no study has exclusively explored the risk factors for PPD in the CS population. As the global rate of CS is expected to increase to 28.5% with more than 38 million CS births in 2030 (10), the risk factors for PPD in women who undergo CS should be identified. Since there are many influencing factors for emergency CS, including maternal and fetal factors, we conducted a prospective study to investigate the risk factors for PPD in women who underwent elective CS before onset of labor.

## Materials and methods

### Study participants

This prospective cohort study is based on the Care for Mother Project, an ongoing longitudinal study conducted to investigate maternal and child health problems at the Shanghai First Maternity and Infant Hospital, which is one of the largest tertiary maternity hospitals in Shanghai, China. After receiving approval from the Institute Ethical Committee (KS18158) and obtaining informed written consent, participants were recruited between December 2020 and September 2021.

The inclusion criteria were as follows: pregnant women aged between 20 and 40 years, singleton with 37–40 weeks of gestation, undergoing elective CS before onset of labor [the indications for elective CS included maternal request, previous uterine scar, placenta accreta spectrum, malpresentation (breech, transverse, and compound presentation) and others], living in Shanghai for more than 5 years, and who participated in the Care for Mother Project and completely answered the questions from an interviewer. The exclusion criteria were as follows: multiple pregnancy, cerebral diseases (e.g., a history of brain surgery or trauma), inability to understand and respond to the questionnaire, chronic pain being managed with pain medications, and prolonged postoperative hospital stay (>4 days).

This study required all women to undergo follow-up during the postoperative period before discharge and 6 weeks after CS. All women suspected of having PPD were advised to consult a psychiatrist.

### Postpartum depression and follow-up

The aim of this study was to investigate the associations of various sociodemographic and clinical factors with the development of PPD at 6 weeks postpartum. The presence of PPD was assessed using the Edinburgh Postnatal Depression Scale (EPDS), which is a 10-item questionnaire. Each question item is graded from 0 to 3, and EPDS score increases with symptom severity. Although a cut-off score of 9 or 10 has been proposed to identify PPD, a score of ≥ 11 may maximize combined sensitivity and specificity (16). Therefore, PPD was defined by a total EPDS score of ≥ 11 at 6 weeks postpartum in this study.

**FIGURE 1:**
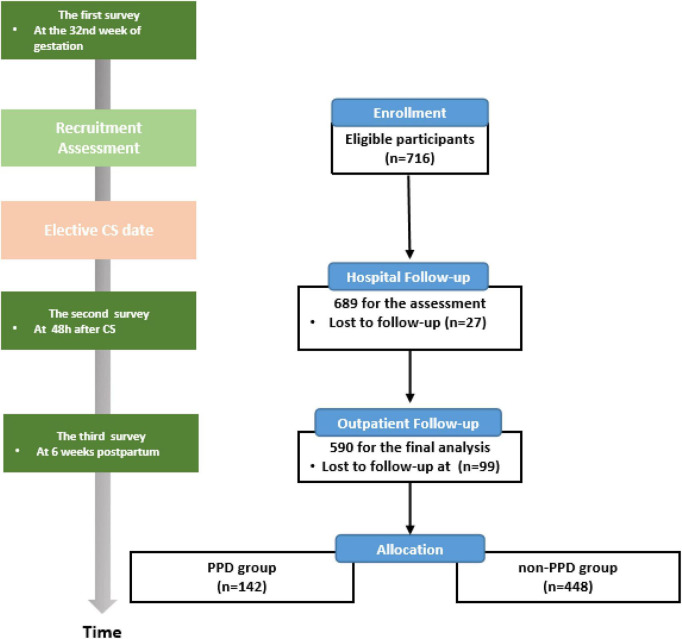
Flow diagram of participant recruitment and the timeline for the three surveys. PPD, postpartum depression; CS, cesarean section.

The participants underwent face-to-face interviews conducted by a trained researcher at three time points: 32nd week of gestation, 2 days postpartum, and 6 weeks postpartum, during which they completed the questionnaires (Figure 1).

In the first survey, baseline information was collected. The demographic information included age, body mass index (BMI), parity, gestational weeks, household income, educational level, history of anxiety/depression, psychological trauma, and chronic pain. Pregnancy-related information included mode of conception (assisted reproductive technology or natural conception) and pregnancy complications (including gestational hypertensive disorders, thyroid diseases, and gestational diabetes mellitus). The participants were requested to complete self-report questionnaires including antenatal self-rating depression scale (SDS), self-rating anxiety scale (SAS), and social support rating scale (SSRS).

Intraoperative variables including anesthesia method, duration of surgery, blood loss, birth weight, Apgar score, and proportion of newborns admitted to the neonatal intensive care unit (NICU) were collected from the electronic anesthesia records. The information gathered in the second survey included satisfaction with anesthesia, EPDS score, pain caused by uterine contraction, and surgical scar. Postpartum pain was defined by an NRS score of ≥ 3 (17, 18).

In the third survey, assessments were performed including pain and EPDS at 6 weeks postpartum. The women were also asked if pain affected their quality of sleep or daily life and if they needed medication treatment. Phone contact was made by a researcher before the survey at 6 weeks. If a participant refused a face-to-face interview, they were considered lost to follow-up.

The Chinese versions of the four questionnaires including EPDS, SDS, SAS, and SSRS used in this study have been well validated and commonly used in perinatal period (19–22).

### Statistical analysis

In descriptive analysis, continuous variables are expressed as mean ± standard deviation and categorical variables as frequency and percentage. Differences in general characteristics between the PPD and non-PPD groups were analyzed using the independent *t*-test or Chi-square test, respectively. All variables with *P*-values of < 0.10 in univariate analysis were included in the following multivariate analysis. Multivariable logistic regression analysis was performed to determine the risk factors for PPD at 6 weeks postpartum. All tests were two-tailed, and the level of significance was set at a *P*-value of < 0.05. All statistical analyses were performed using the software package SPSS, version 18.0.

### Sample size

In this prospective cohort study, we used multivariate logistic regression to identify the risk factors for PPD out of 34 variables (see Tables 1, 2). According to the classic methodological paper on the simulation study of the number of events per variable (EPV) in logistic regression analysis, no major problem occurred in the parameter estimates and usual tests of significance in the logistic model when EPV values of 10, 15 or greater (23). So, in our study, the total samples of 340–510 are enough to guarantee the adequate significance of the results. We enrolled 590 women for final analysis.

**TABLE 1:** Demographic and pregnancy-related characteristics among women included in the study.

Characteristics	PPD (*n* = 142)	Non-PPD (*n* = 448)	*P*-value
**Demographic**			
Age^b^, years	31.6 (4.4)	32.4 (5.3)	0.10
Body mass index before pregnancy^b^, kg⋅m^–2^	21.8 (3.7)	23.3 (14.4)	0.29
Body mass index during pregnancy^b^, kg⋅m^–2^	26.7 (3.9)	26.7 (4.3)	0.22
Gestational weeks^b^	38.4 (0.8)	38.4 (1.0)	0.91
Parity	0 (0, 1)	0 (0, 1)	0.96
**Level of education^a^**			
≤ Elementary education	0 (0)	0 (0)	0.10
Secondary education (junior/senior)	14 (9.8)	44 (9.8)	
Undergraduate education	114 (80.3)	321 (71.7)	
Postgraduate education	14 (9.9)	83 (18.5)	
**Monthly household income per capita^a^, ¥**			
< 2,000	1 (0.7)	3 (0.7)	0.94
2,000–4,999	4 (2.8)	14 (3.1)	
5,000–9,999	47 (33.1)	160 (35.7)	
≥ 10,000	90 (63.4)	271 (60.5)	
Self-reported history of anxiety/depression^a^	4 (2.8)	5 (1.1)	0.23
Self-reported history of psychological trauma^a^	3 (2.1)	3 (0.7)	0.15
Self-reported history of chronic pain^a^	2 (1.4)	5 (1.1)	0.68
**Indications for elective cesarean** ^a^			
Maternal request	61 (43.0)	203 (45.3)	0.35
Previous uterine scar	50 (35.2)	138 (30.8)	
Placenta accreta spectrum	14 (9.9)	29 (6.5)	
Malpresentation	10 (7.0)	46 (10.3)	
Others	7 (4.9)	32 (7.1)	
**Pregnancy-related variables**			
Assisted reproductive technology^a^	14 (9.9)	37 (8.3)	0.68
**Pregnancy complications^a^**			
Hypertension	7 (4.9)	29 (6.5)	0.64
Gestational diabetes mellitus	17 (12.0)	59 (13.2)	0.82
Thyroid disease	10 (7.0)	35 (7.8)	0.91
Antenatal SSRS^b^	46.5 (6.3)	46.7 (5.8)	0.74
Antenatal SDS^c^	33.0 (12.0)	31.0 (8.0)	<0.001*
Antenatal SAS^c^	33.0 (7.3)	30.0 (6.0)	<0.001*

PPD, postpartum depression; SSRS, Social Support Rating Scale; SDS, Self-Rating Depression Scale; SAS, Self-rating Anxiety Scale.

^a^For n (%); ^b^for Mean (SD); ^c^for Median (IQR).

*P < 0.05.

**TABLE 2:** Distribution of the study participants by intraoperative and postpartum characteristics.

	PPD (*n* = 142)	Non-PPD (*n* = 448)	*P*-value
**Intraoperative variables**			
Repeated CS^a^	48 (33.8)	134 (29.9)	0.44
Anesthesia method^a^			
General anesthesia	0 (0)	7 (1.6)	0.17
CSEA	141 (99.3)	439 (98.2)	
Epidural anesthesia	1 (0.7)	1 (0.2)	
Duration of surgery^b^, min	41.3 (15.4)	40.0 (11.9)	0.29
Blood loss during the surgery^b^, ml	325.8 (88.6)	317.7 (57.9)	0.22
Neonatal birth weight^b^, g	3371.5 (444.3)	3376.8 (399.7)	0.89
Apgar score at 1 min^b^	9.3 (0.5)	9.4 (0.7)	0.12
Apgar score at 5 min^b^	9.9 (0.3)	9.9 (0.3)	0.91
Newborns admitted to the NICU^a^	2 (1.4)	5 (1.1)	0.68
**Postpartum variables at 2 days**			
Satisfaction with anesthesia^b^	9.3 (1.3)	9.4 (1.3)	0.78
Pain of uterine contraction^a^	96 (67.6)	257 (57.4)	0.03*
NRS for pain of uterine contraction^b^	2.8 (2.6)	2.6 (2.8)	0.30
Pain from surgical scar^a^	116 (81.7)	329 (73.4)	0.047*
NRS for pain from surgical scar^b^	3.4 (2.4)	2.9 (2.4)	0.03*
EPDS score^c^	4.0 (5.0)	3.0 (3.0)	0.005*
Postpartum depressive symptoms (EPDS ≥ 11)^a^	20 (14.1)	5 (1.1)	<0.001*
**Postpartum variables at 6 weeks**			
Pains at 6 weeks postpartum^a^	70 (49.3)	122 (27.2)	<0.001*
Pain that interfered with daily life	38 (26.8)	57 (12.7)	<0.001*
Pain that affected the quality of sleep^a^	25 (17.6)	36 (8.0)	0.001*
Need for pain medication^a^	12 (8.5)	18 (4.0)	0.036*

PPD, postpartum depression; CS, cesarean section; CSEA, Combined spinal-epidural anesthesia; NICU, neonatal intensive care unit; NRS, Numeric Rating Scale; EPDS, Edinburgh Postnatal Depression Scale.

^a^For n (%); ^b^for Mean (SD); ^c^for Median (IQR).

*P < 0.05.

## Results

### Baseline characteristics of the participants

Of the recruited 716 participants who completed the prenatal baseline questionnaire, 689 (96.2%) completed the second survey in the hospital and 590 (82.4%) underwent a face-to-face interview at 6 weeks postpartum. The lost-to-follow-up rate was 17.6% (Figure 1).

Among the 590 women, PPD (EPDS score ≥ 11) was identified in 142 women (25.4%). The baseline characteristics of the PPD and non-PPD groups are summarized in Table 1. The average age of the PPD group was slightly lower than that of the non-PPD group (31.6 vs. 32.4 years). Parity, gestational weeks, BMI before and during pregnancy, educational level, monthly household income and the indications for elective CS were similar between the groups. There were no differences in self-reported history of anxiety or depression, psychological trauma, chronic pain, assisted reproductive technology, and pregnancy complications (hypertension, gestational diabetes mellitus, and thyroid disease) between the groups. Compared with the non-PPD group, the PPD group had higher antenatal SDS and SAS scores (33.0 vs. 31.0 and 33.0 vs. 30.0, *P* < 0.001), whereas there was no significant difference in antenatal SSRS score between the groups.

### Intraoperative and postpartum characteristics

Intraoperative and postpartum characteristics are displayed in Table 2. More women in the PPD group complained about incision pain and uterine contraction pain than those in the non-PPD group at 2 days postpartum. Until the 6th week postpartum, 49.3 and 27.2% of the women reported pain in the PPD and non-PPD groups, respectively. More PPD women complained that their daily life and sleep were affected by pain and required medication to relieve the pain. Interestingly, the EPDS score at 2 days postpartum was higher in the PPD group, which also had a higher proportion of those with an EPDS score of ≥ 11 before discharge, than in the non-PPD group. Repeated CS was reported in 33.8 and 29.9% of women in the PPD and non-PPD groups, respectively. No significant differences were observed between the groups in the following variables: anesthesia method, duration of surgery, blood loss, neonatal birth weight, Apgar score, number of newborns admitted to the NICU, and satisfaction with anesthesia.

### Association of some risk factors with postpartum depression as identified by multiple logistic regression analysis

The adjusted OR for various factors associated with an EPDS score of ≥ 11 calculated using multivariable binary logistic regression is shown in Table 3. The risk factors associated with PPD in multivariate analysis were a high antenatal SAS score (OR = 1.10, 95% CI = 1.04–1.16), postpartum depressive symptoms at 2 days postpartum (OR = 6.17, 95% CI = 1.35–28.31), and pain at 6 weeks postpartum (OR = 2.14, 95% CI = 1.24–3.69).

**TABLE 3:** Risk factors for PPD in women who underwent elective CS.

Characteristics	Unadjusted OR (95%CI)	*P*-value	Adjusted OR (95%CI)	*P*-value
Antenatal SDS	1.06 (1.03–1.09)	<0.001*	1.00 (0.96–1.05)	0.93
Antenatal SAS	1.13 (1.09–1.18)	<0.001*	1.10 (1.04–1.16)	0.002*
Pain of uterine contraction at 2 days postpartum	1.55 (1.04–2.31)	0.031*	1.33 (0.86–2.06)	0.19
Pain from surgical scar at 2 days postpartum	1.61 (1.00–2.59)	0.048*	2.06 (0.98–4.34)	0.056
NRS for pain from surgical scar at 2 days postpartum	1.08 (1.00–1.17)	0.034*	0.97 (0.85–1.10)	0.65
EPDS at discharge	1.18 (1.11–1.26)	<0.001*	1.04 (0.94–1.16)	0.44
Postpartum depressive symptoms at 2 days postpartum (EPDS ≥ 11)	14.53 (5.34–39.49)	<0.001*	6.17 (1.35–28.31)	0.019*
Pain at 6 weeks postpartum	2.60 (1.76–3.83)	<0.001*	2.14 (1.24–3.69)	0.006*
Pain that interfered with daily life	2.51 (1.58–3.99)	<0.001*	1.35 (0.66–2.80)	0.41
Pain that affected the quality of sleep	2.45 (1.41–4.24)	0.001*	1.01 (0.45–2.25)	0.98
Need for pain medication	2.21 (1.04–4.70)	0.04*	0.99 (0.39–2.56)	0.99

OR, odds ratio; CI, confidence interval; SDS, Self-Rating Depression Scale; SAS, Self-rating Anxiety Scale; NRS, Numeric Rating Scale; EPDS, Edinburgh Postnatal Depression Scale.

*P < 0.05.

## Discussion

The main findings of this study suggested that high antenatal SAS scores, PPD symptoms (EPDS ≥ 11) at 2 days postpartum, and pain at 6 weeks postpartum were independently associated with PPD.

CS rates have been increasing worldwide with different effects on maternal and neonatal health. The association between CS and PPD also raises concern. A previous meta-analysis including 32 articles demonstrated that CS and emergency CS were significantly related to an increased risk of PPD (24). However, there seems to be no study that has assessed the risk factors for PPD in the population that underwent CS. Since emergency CS is performed for multifarious medical reasons and increases perioperative complications (25), we only enrolled women who underwent elective CS in the present study.

In total, 142 (25.4%) participants had PPD. In a recent study, PPD was reported in 19.8% (43/217) and 15.4% (47/305) of Chinese women who underwent cesarean and vaginal delivery, respectively (26). In another study from Japan, the incidence of PPD were 13.4% (30/224) in the CS group and 7.1% (77/1086) in the vaginal delivery group (9). The difference in the reported incidence of PPD may be related to the EPDS cut-off score used in different studies. EPDS is the most commonly used depression screening tool for major depression in postpartum women. A recent meta-analysis revealed that an EPDS cut-off score of ≥ 11 maximized combined sensitivity (0.81) and specificity (0.88) to detect PPD (16). Therefore, an EPDS score of ≥ 11 may be the optimal cut-off for maternal depression (27). Accordingly, PPD was defined by an EPDS score of ≥ 11 at 6 weeks postpartum in this study. Our cut-off score was higher than those in the first two studies (9, 26), and the incidence of PPD in this study was higher than those reported in those studies. One possible reason is that maternal PPD was assessed in different postpartum periods from 1 to 3 months.

The prevalence of prenatal anxiety and depression symptoms is very common during pregnancy. Approximately 10–25% of pregnant individuals are typically affected by anxiety (6, 28), which is related to a higher risk of both maternal and child health. Poorer child’s sleep has been reported to be significantly associated with maternal prenatal anxiety (29), which may in turn influence mother’s sleep and mood. A large prospective cohort study found that prenatal anxiety and depression were key risk factors for PPD (5). Cheng et al. (30) also indicated that prenatal anxiety could predict PPD symptoms. In this study, we used antenatal SDS and SAS to assess depression and anxiety status at 32 weeks of gestation. High SDS and SAS scores were associated with PPD in the univariate analysis, but only SAS remained significant when adjusted with all other variables. Consequently, we suggest that elevated prenatal anxiety symptom is a prenatal risk factor for PPD. A study from Italy also found that women with anxiety showed a significant tendency to PPD (31). Our study was conducted during the coronavirus (COVID-19) pandemic. Women giving birth during the pandemic may suffer more anxiety and depression. Pandemic sequelae such as fear of infection, face mask mandates, women with CS perceiving visitation restrictions, and social isolation may increase anxiety, which can contribute to PPD (32, 33).

In particular, full assessment for depression and anxiety before CS is suggested, just as recommended by the American College of Obstetricians and Gynecologists that depressive and anxiety symptoms should be screened in pregnant women at least once during pregnancy and postpartum (34). A study explored trends of stress and anxiety symptoms from pregnancy to postpartum and found that the intensity of anxiety significantly increased at 36 weeks of gestation, especially when close to delivery (30). According to our results, active management of stress and anxiety should be provided at least before cesarean delivery.

Generally, satisfaction with social support moderates stress and anxiety in pregnant women (35), and low social support is a risk factor for PPD (36). We assessed social support using the SSRS, which includes subjective social support, objective social support, and utilization of support. The total score is the sum of the three dimensions, with an aggregate score ranging from 7 to 56. However, we did not evaluate the difference in SSRS score between the PPD and non-PPD groups. In this study, all women in two groups had high SSRS scores (mean 46.5 and 46.7), with A higher score indicating higher levels of social support (37). Therefore, SSRS score was not found to be a risk factor for PPD in this study.

This study demonstrated that women with PPD reported higher EPDS scores and depressive symptoms (EPDS ≥ 11) than women without PPD at 2 days postpartum. An early postoperative EPDS score of ≥ 11 was associated with PPD at 6 weeks postpartum. EPDS is commonly applied to screen PPD symptoms after 4 weeks postpartum. Since PPD treatment is difficult, detection and interventions need to be conducted as early as possible. Therefore, a question was raised regarding the possibility of identifying women at risk of PPD in the immediate postpartum period using the EPDS. A longitudinal study was conducted to evaluate the EPDS scores of patients at 1, 4 and 8 weeks postpartum. A high EPDS score in the 1st week was predictive of the risk of elevated EPDS scores and PPD symptoms at 4 and 8 weeks postpartum (38). Another study included women who underwent vaginal delivery or CS and completed the EPDS questionnaire 2 days postpartum. Total EPDS score was higher in women who underwent CS than in those who underwent vaginal delivery (2). The authors believed that the EPDS score in the early postpartum period could identify depressive symptoms following delivery. Consistent with previous findings, we also found that 2-day EPDS scores closely reflected 6-week scores postpartum. Early intervention preventing PPD should be facilitated for the women with high EPDS scores 2 days after CS.

Despite finding that women with PPD had more severe pain from uterine contraction and incision in the acute postoperative period, only the pain at 6 weeks postpartum was associated with the risk of PPD development. More women in the PPD group reported that their daily life and quality of sleep were affected by the pain and they needed analgesic medicine at 6 weeks after CS.

Approximately one-fifth of women who undergo CS experience severe pain in the early postoperative stage, increasing the risk of developing chronic pain and PPD (39). A meta-analysis including 38 studies reported an overall 15.4% of women experiencing persistent wound pain between 3 and 6 months after CS (40). It is likely that the acute postoperative pain is associated with the development of persistent cesarean scar pain (41). According to a longitudinal cohort study, the prevalence of severe acute pain was 10.9% within 36 h postpartum and that of persistent pain was 9.8% after cesarean or vaginal delivery (42). The study suggested that severe acute postpartum pain was independently related to the risk of persistent pain and PPD (42). In our study, the severity and incidence of acute and persistent pain were higher in the PPD group than in the non-PPD group. Persistent pain may have negative effects on activities of daily living, sleep, breastfeeding, and newborn care, which contribute to the onset of PPD (40, 43). Therefore, we suggest that acute pain should be well managed during the first few days after CS to prevent longer-term morbidities.

For untreated maternal depression during the pre- and post-natal period has been associated with adverse outcomes in both mother and baby, the pharmacological and non-pharmacological measures to treat the perinatal depression are very important. Several antidepressants seem to be safe during pregnancy and postpartum, but they should be weighed against the risk/benefit by clinicians (44). Brexanolone was firstly approved by the US Food and Drug Administration for the treatment of patients with PPD in recent years (45). In the future, many studies comparing brexanolone and conventional antidepressants are still needed. Non-pharmacological measure should also be emphasis. Avoiding angry and administrating more social support may help improve maternal mood disorders (46).

### Strengths and limitations

The strengths of the study are as follows: First, this study only enrolled women who underwent elective CS, avoiding the influence of delivery mode and emergency CS. In addition, the questionnaires were administered by face-to-face interview at 2 days and 6 weeks postpartum, eliminating bias in communication, while the postnatal questionnaires were completed by follow-up telephone calls in many previous studies.

However, some limitations should be considered when interpreting the results. First, data were collected from a single institution in China, which limits the generalization of the findings. Second, 16.6% of the participants were not included in the analysis because they refused to complete the questionnaire in the face-to face survey. Third, the reason for refusing the interview at 6 weeks postpartum should be investigated, for the lost to follow-up may have some impact on the results. But most of them refused to explain the reason not to receive the interview on the phone. Lastly, previous studies indicated that breastfeeding may offer protective benefits against PPD (47, 48). We did not explore the effects of breastfeeding on maternal PPD. The item of pain interfering with daily life in our questionnaire only assessed the effects of pain on breastfeeding.

## Conclusion

This study suggests that prenatal anxiety, postnatal factors including early elevated EPDS score and persistent pain have significantly positive association with the risk of PPD in women who underwent elective CS, implying that effective interventions should be considered to address individual risk factors. Further evidence is required to explore the associations between specific factors and the risk of PPD in emergency CS.

## Data availability statement

The raw data supporting the conclusions of this article will be made available by the authors, without undue reservation.

## Ethics statement

The studies involving human participants were reviewed and approved by the Ethics Committee of Shanghai First Maternity and Infant Hospital, School of Medicine, Tongji University, Shanghai. The patients/participants provided their written informed consent to participate in this study.

## Author contributions

RL and YL conducted the study and wrote the manuscript draft. WL and BZ helped conduct the study and analyze the data. ZL contributed to the study design and made critical revisions to the article. ZX conducted the study, supervised its design, and was responsible for the analysis and interpretation of data. All authors contributed to the article and approved the submitted version.

## Abbreviations

PPD: postpartum depression
CS: cesarean section
EPDS: Edinburgh Postnatal Depression Scale
SSRS: Social Support Rating Scale
SDS: self-rating depression scale
SAS: self-rating anxiety scale
BMI: body mass index.
